# Supplementary material for: Low bone mass in people living with HIV on long-term anti-retroviral therapy: A single center study in Uganda
Source: PLoS One. 2021 Feb 5;16(2):e0246389. doi: 10.1371/journal.pone.0246389 (PMC7864439; doi:10.1371/journal.pone.0246389)
Supplement: Attachment — Submitted filename: Response to reviewer comments_R3.docx [file pone.0246389.s002.docx]

Dear Editor,

We would like to thank you for reviewing our manuscript to ensure that is it worth publishing in PLOS One. Below is a point by point response to the comments.

**Comment:** We note that you have included tables 1,2 and 4 but there is not Table 3, if this should be included then please include a copy within your manuscript and cite in your text. - or. If table 4 should actually be Table 3 then please amend the numbering of the table and citations within your text

**Response**: Thanks very much for this observation. Table 3 was mislabeled as Table 4. This has been rectified. “Table 4” now appears as “Table 3”.

**Comment:** Please amend the title either on the online submission form or in your manuscript so that they are identical.

**Response:** This has been done
