# Supplementary material for: Low bone mass in people living with HIV on long-term anti-retroviral therapy: A single center study in Uganda
Source: PLoS One. 2021 Feb 5;16(2):e0246389. doi: 10.1371/journal.pone.0246389 (PMC7864439; doi:10.1371/journal.pone.0246389)
Supplement: Attachment — Submitted filename: Response to reviewer comments.docx [file pone.0246389.s001.docx]

**Response to reviewers**

**Reviewer #1**

**Comment:** *The survey does give the general picture of BMD among a well-	represented 200 PLWD. Since only one BMD was taken, the data did	not provide	information	about the trend of development, particularly	among the group with low	BMD. To give real clinical value to the survey,	another BMD needs to be	arranged in	2-3 years' time to identify the changes so as to allow some practical	recommendations on the	management*

**Response:** We are very interested in longitudinal follow up of the bone health of these individuals and are therefore working on a grant application to include bone health as one of the routine annual follow up procedures for the cohort. If successful we hope to follow them up for up to 96 weeks.

**Reviewer #2**

**Comment:** *How were the participants selected? What were the inclusion criteria? A consort flow diagram would be very helpful*.

**Response:** We have added a statement “Using computer generated numbers we randomly enrolled adults above 18 years who had been receiving ART for at least 10 years and were willing and able to comply with all study procedures” Lines 87-89. We have also added a flow diagram as suggested.

**Comment:** *Ref 22/23 are not appropriate: most participants were under 50 years old. Therefore, the ISCD (not the WHO!) recommends to apply the Z-scores, not the T-scores. Low bone mass is defined as a Z-scores <-2.0 meaning that long-term ART is not associated with low BMD. The categorisation of the measurements should be provided: how many participants had actually a Z-score below -2.0?*

**Response:** We have re-analysed the data using the official position of the International Society of Clinical Densitometry (ISCD) since most participants were below 50 years of age. A Z-score of -2.0 or lower has been defined as having low bone mass for age and a Z-score above -2.0 has been defined as bone mass within the expected range for age. The World Health Organization has been used for post-menopausal females and males above 50 years of age. For ease of data analysis, all participants with low bone mass or osteopenia/osteoporosis have been categorized as having “low bone mass” while the rest are categorized as having normal bone mass (BM). The term “Bone Mass” has been adopted in place of “Bone Mineral Density”. 4/199 and 36/199 have either Z-scores below -2.0 or T-score below -1.0 for the hip and lumbar spine respectively.

The title has also been changed to *“Low bone mass in people living with HIV on long-term anti-retroviral therapy in Uganda”*

**Comment:** *discussion: the recommendations for screening are not supported by the data shown*.

**Response:** The recommendation on screening has been deleted.

**Comment:** *conclusion: please do not over interpret our findings: association does not mean causality!*

**Response:** The conclusion has been recast.

**Reviewer #3**

**Comment*:*** *Abstract: "This study set out to determine the prevalence and predictors of low BMD following long-term exposure to antiviral therapy....".
It seems to me that you have evaluated the prevalence of low BMD in your study but the predictors of low BMD were only studied in a very limited manner as there was no inclusion of lifestyle, exercise levels, education level, socio-economical and comorbidity, etc. The original statement needs revision to describe your completed work.*
**Response:** This has been revised to *“This study set out to determine the prevalence of low bone mass following long-term exposure to antiretroviral therapy in Ugandan people living with HIV”.*

**Comment:** *The sampling: need more description on random or convenient sampling from your pool of 8000.*

**Response:** A better description is given and a flow diagram included (Fig 1).

**Comment:** *Table 1: need to include Alcohol data in your disclosure. For the BMI definition of overweight and obese vary between different countries, suggest to list out clearly.*

**Response:** A statement had been added with appropriate references ***“****The WHO general cut off points for BMI classification were used for this study however literature suggests that they may vary with race/ethnicity and between countries”.*

**Comment:** *Table 3: suggest to add a bar chart presentation for clarity.*

**Response:** Table 3 has been replaced by a bar chart (Fig 2).

**Comment:** *Table 4: need to improve the presentation, plus add more explanation and justification*

**Response:** More information has been provided in the narrative to ease interpretation of Table 4 (Now Table 3).

**Comment: 6.** *Having revised 1 to 5, the title of the paper could be modified to reflect your actual completed work.*

**Response:** The title has been revised to “*Low bone mass in people living with HIV on long-term anti-retroviral therapy in Uganda”.*
